# Social determinants of youth with mild intellectual disability in outpatient care for mental health problems: a case-control study

**DOI:** 10.1192/j.eurpsy.2025.1177

**Published:** 2025-08-26

**Authors:** M. Storm, E. J. Giltay, R. R. Vermeiren, W. M. van Eldik, E. C. Palstra, E. D. van Duin, D. van den Berg

**Affiliations:** 1 LUMC, Leiden; 2 AMC, Amsterdam, Netherlands

## Abstract

**Introduction:**

Children with mild intellectual disability (MID) face specific challenges threatening their development, particular their mental health. They face a heightened risk of psychopathology (Buckley et al. ANZJP 2020; 54 970–984). This heightened susceptibility is theorized to be shaped by a complex interplay of diverse socio-demographic factors experienced by these children, collectively known as social determinants of mental health (SDOMH), include ethnicity, socioeconomic status, household conditions, family dynamics, and neighborhood deprivation (Allen et al. IJP 2014; 26 392–407).

**Objectives:**

This study examined the collective and unique role of diverse social determinants of mental health (SDOMH) associated with mental health problems (MHP) for children with MID, compared to peers with and without MHP.

**Methods:**

Combining microdata from Statistics Netherlands, municipality data from The Hague and mental health care records, this population-based case-control study included four groups aged 0-17 years (*M*_age_ = 10.6, 35.6% female). Two groups of children receiving outpatient mental health care for MHP with MID (*n*=505) and without MID (*n*=2,767), each with a matched control group from the general population (*n*=2,525 and *n*=13,835, respectively), were studied. Through multivariate logistic regression analyses, both MHP groups were compared to their control group and each other to examine the likelihood of a particular SDOMH associated with receiving mental health care for MHP in children with and without MID.

**Results:**

Children with MID receiving mental health care showed significant differences in multiple domains compared to their control group and to children receiving mental health care without MID. In the multivariate model, they were less likely to have European-born mothers, more likely to have parents with moderate or low education levels, and tended to live in smaller, single-parent, lower-income households. Similar, though less deviant, patterns were observed for children without MID receiving mental health care compared to the general population, except for parental education. Additionally, children without MID were more likely than their controls to reside in densely populated areas with lower neighborhood education levels.

**Conclusions:**

Our study highlights that diverse SDOMH are associated with the likelihood of receiving care for MHP in children. Moreover, children with MID face disproportionate disadvantages, particularly regarding low parental education and household income. Thus, interventions should not only target the child but also their family and environmental context.

**Disclosure of Interest:**

None Declared

